# Mitogenomic Analysis and Conservation Genetics of the Endangered Oriental Stork (*Ciconia boyciana*)

**DOI:** 10.3390/ani16071077

**Published:** 2026-04-01

**Authors:** Xiao-Die Chen, Yun-Yun Wang, Zhi-Min Xu, Lin Xiao, Chang-Hu Lu, Cheng-He Sun, Cheng-Zhi Li

**Affiliations:** 1 College of Life Sciences, Nanjing Forestry University, Nanjing 210037, China; chenxiaodie5206@163.com (X.-D.C.); luchanghu@njfu.com.cn (C.-H.L.); 2 Sihong Hongze Lake Wetland National Nature Reserve, Suqian 223900, China; 115313380@163.com (Y.-Y.W.); xuzhimin24@126.com (Z.-M.X.); xl18556242118@163.com (L.X.)

**Keywords:** endangered species, structural conservation, genetic comparison, nucleotide sequence

## Abstract

Despite the endangered status of the Oriental Stork (*Ciconia boyciana*) on the IUCN Red List, a critical lack of contemporary mitochondrial genomic data from its core distribution areas in mainland China has hindered our understanding of the species’ long-term evolutionary stability and spatiotemporal variation. This study addresses this gap by sequencing and assembling the complete mitogenome (17,608 bp) of a contemporary individual from Hongze Lake, Jiangsu (PX682155), and conducting a rigorous comparative analysis against a historical reference sequence published 25 years ago in Japan (NC_002196). Our results demonstrate striking structural and functional conservation across a quarter-century span; the 13 protein-coding genes exhibit highly synchronized gene arrangements, base composition biases, and Relative Synonymous Codon Usage (RSCU) patterns, indicative of stringent purifying selection maintaining oxidative phosphorylation efficiency. While phylogenetic analysis reinforces its sister-group relationship with the White Stork (*C. ciconia*), significant length polymorphisms were identified within the D-loop control region, primarily driven by microsatellite repeat variations. These findings provide a vital genomic benchmark for mainland populations, offering high-resolution molecular markers essential for future large-scale assessments of geographic isolation and the refinement of targeted conservation strategies for this flagship wetland species.

## Simple Summary

The Oriental Stork (*Ciconia boyciana*) is a rare wetland bird currently listed as Endangered on the IUCN Red List. While it is a flagship species for conservation, there has been a significant lack of up-to-date mitochondrial genomic data from its core habitats in mainland China. To address this, we sequenced the complete mitochondrial genome of a contemporary stork from Hongze Lake, Jiangsu (collected in 2025), and compared it with a historical reference sequence from Japan published 25 years ago. Our analysis shows that the species’ genetic structure and function have remained remarkably stable over the past quarter-century. However, we identified specific differences in the length of the “D-loop” region, which are caused by variations in repeating DNA sequences. These findings establish a vital new genomic benchmark for the mainland population. By providing high-resolution molecular markers, this research helps scientists better monitor geographic isolation and develop more effective, targeted conservation strategies to protect this endangered species across its migratory routes.

## 1. Introduction

The Oriental Stork (*Ciconia boyciana*), a distinguished member of the family Ciconiidae within the order Ciconiiformes, is currently listed as Endangered (EN) on the IUCN Red List and designated as a Class I National Key Protected Wild Animal in China. Morphologically, it is a majestic large wading bird characterized by its pure white plumage contrasted against black primary and secondary flight feathers, and a distinctive, heavy black bill that tapers to a point. Unlike the closely related White Stork (*C. ciconia*), the Oriental Stork possesses a notable patch of vermilion bare skin around its eyes and pale iris coloration, which, combined with its long, bright red legs, makes it an unmistakable figure in its natural habitat. As a specialized wetland dweller, this species exhibits extreme sensitivity to water clarity, vegetation coverage, and prey density, particularly fish and small amphibians. Consequently, it is regarded as a flagship “indicator species” for assessing the health of wetlands along the East Asian–Australasian Flyway (EAAF) [1,2]. Historically, the species maintained a robust distribution across China, Russia, Japan, and the Korean Peninsula. However, beginning in the mid-20th century, wild populations plummeted due to anthropogenic pressures, including habitat loss from intensive agricultural expansion, persistent environmental pollution, and illegal poaching [3]. Although intensive conservation efforts—such as artificial reintroduction programs and the rigorous establishment of nature reserves—have catalyzed a recent population rebound, the global census remains precariously low at approximately 3000 to 4000 individuals. Furthermore, the extant population exhibits concerning trends of fragmentation and localization, posing severe challenges to the long-term maintenance of genetic diversity and the evolutionary resilience of the species [4,5].

The migration of Oriental Stork is a typical seasonal long-distance migration, with its trajectory deeply embedded in the geographical framework of EAAF. On a global scale, these birds depart their breeding grounds in the Russian Far East and Northeast China each spring, following two southward routes: the western route passes through the eastern foothills of the Greater Khingan Mountains and crosses the Bohai Rim to reach the Yellow River Delta, while the eastern route descends along the Sanjiang Plain and moves toward the Chinese coast via the western Korean Peninsula [6]. Within China, these “national treasure” birds exhibit highly concentrated belt-like migration tracks, primarily utilizing coastal wetland corridors or the inland Songliao and North China Plains to migrate south, eventually overwintering in clusters around Poyang Lake, Dongting Lake, and various lakes in Anhui Province within the middle and lower reaches of the Yangtze River [7]. As a flagship species of the EAAF, its migratory behavior demonstrates significant ecological adaptability and population differentiation: contemporary Chinese populations exhibit two coexisting overwintering patterns—long-distance migration from the Heilongjiang River basin to the middle and lower Yangtze plains, and short-distance migration staying within the Bohai Rim and North China Plain.

Current research on Oriental Stork has established a multi-dimensional framework encompassing population dynamics, habitat ecology, and conservation biology. Specifically, scholarly focus has centered on population fluctuations during wintering and breeding seasons, habitat utilization patterns [8,9], changes in population size and genetic diversity of captive and reintroduced populations [10,11], comprehensive conservation strategies for this endangered flagship species [12,13], suitability assessments of potential habitats [14,15], and the intricacies of migratory routes and stopover strategies [16,17]. As a rare and endangered bird, genetic research is fundamental to revealing adaptive evolution, population genetic structure, and formulating effective conservation strategies. Recent advancements include the development of a PCR-based sex identification method using single feathers by Itoh et al., providing non-invasive and rapid technical support for this monomorphic species [18]. Molecular evidence has traced the origin of the species; microsatellite markers identified by Huang et al. clarified genetic variation and gene flow within populations [19,20]; and comparative studies on gut microbiota revealed significant differences between captive and wild individuals, providing physiological bases for evaluating rewilding feasibility [21]. Furthermore, Yang et al. achieved the first high-quality, chromosome-level genome assembly of Oriental Stork, filling a critical gap in genomic resources [22]. These studies have constructed a core framework for Oriental Stork genetic biology—spanning sex, diversity, population structure, and adaptation—providing vital scientific support for species recovery. In conservation practice, identifying genetic boundaries and the evolutionary history of different geographical populations is central to precise strategy formulation. As early as 2000, Japanese researchers completed the first mitogenome sequencing of the Oriental Stork, laying the foundation for molecular phylogenetics [23]. A quarter-century later, amidst habitat changes driven by global climate shifts, there is an urgent need to determine whether significant genetic differentiation exists between Japanese and mainland populations, and how such differentiation is reflected in mitochondrial coding and non-coding regions. Currently, a substantial gap remains in the mitogenomic data for mainland Chinese populations, which serve as the primary breeding and wintering grounds. In particular, the local population in Hongze Lake, Jiangsu, located at a critical ecological node in the lower Huai River wetlands and exhibiting unique “local migration” habits, may have evolved a distinct genetic lineage through long-term geographic isolation [4].

Therefore, this study successfully assembled the complete mitochondrial genome of an Oriental Stork sampled from Hongze Lake, Jiangsu (2025), utilizing high-throughput sequencing and de novo bioinformatic pipelines. By conducting a systematic spatiotemporal comparative genomic analysis against the Japanese reference sequence from 2000, we aim to elucidate the structural characteristics and gene arrangement patterns of the East Asian mainland population. The research focuses on characterizing the nucleotide substitution patterns of protein-coding genes (PCGs), identifying length polymorphisms in the hypervariable D-loop, and reconstructing the phylogenetic position of the mainland lineage. This work fills a critical gap in the mitogenomic data for mainland populations and provides a high-resolution molecular framework for evaluating the genetic connectivity and divergence between island and continental populations over a 25-year interval.

## 2. Materials and Methods

### 2.1. Sample Collection and Genomic Sequencing

**Figure 1:**
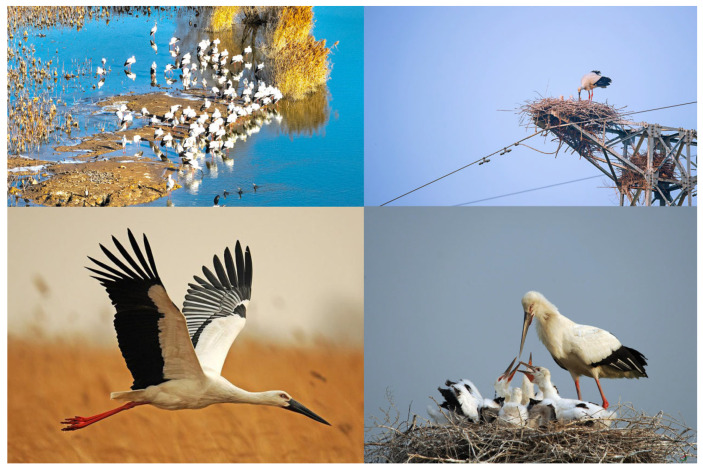
Overwintering and breeding individuals of the Oriental Stork (*Ciconia boyciana*) in the Hongze Lake Nature Reserve, Jiangsu.

In April 2025, peripheral blood samples were collected from four wild nestlings of Oriental Stork in Suqian, Jiangsu Province. All individuals were in healthy condition, though their sexes were undetermined. For this study, one high-quality sample was selected for sequencing to represent the Chinese population in comparison with the Japanese lineage (Figure 1). Following collection, the tissue was aliquoted into sterile 1.5 mL microcentrifuge tubes and immediately cryopreserved at −80 °C to maintain genomic integrity. Total DNA extraction and high-throughput sequencing were conducted using equipment from Nanjing Personalbio Technology Co., Ltd. (Nanjing, China) for the generation of high-quality genomic resources. A specialized sequencing library with an approximate insert size of 350 bp was constructed and subsequently processed on the Illumina NovaSeq 6000 platform (San Diego, CA, USA). The sequencing was executed using a Paired-End 150 bp (PE150) strategy on the Illumina platform, yielding approximately 8 × 10 Gb of raw data, ensuring high base-calling accuracy and structural integrity. To facilitate downstream comparative analysis, the complete mitogenome sequence of Oriental Stork (NC_002196) was downloaded from the NCBI GenBank database. This reference sequence provided a template for the architectural annotation and comparative analysis of the newly assembled mitogenome (PX682155). This approach ensured consistency in gene boundary identification and enabled a robust assessment of spatiotemporal genetic variations.

### 2.2. Mitogenome Assembly, Annotation, and Analysis

Raw sequencing data underwent quality control using Fastp v.0.19.7 [24] to remove adapter sequences and filter out highly repetitive sequences, reads with excessive N-base ratios, and low-quality reads. To ensure assembly accuracy, both map-to-reference and de novo assembly were performed. The de novo assembly using MITOZ yielded a scaffold of 17,600 bp, which was consistent with the result from Geneious Prime 2025 [25] (identity > 99.9%). Approximately 99.9% of the total reads were successfully mapped to the assembled mitogenome, confirming the representation of the data. Sequence alignment was performed in MEGA 11 [26], and the mitogenome was visualized using the MITOS web server. To analyze genomic base composition, MEGA 11 was first used to calculate the content of each nucleotide. Nucleotide composition bias was analyzed using the formulas for AT-skew (AT-skew) = (A − T)/(A + T)) and GC-skew (GC-skew) = (G − C)/(G + C)) [27]. Additionally, PhyloSuite v2.0 [28] was utilized to calculate Relative Synonymous Codon Usage (RSCU) and generate corresponding visualization profiles. Simple Sequence Repeats (SSRs) were identified using MISA (Microsatellite identification tool) with thresholds of 10, 5, 4, 3, 3, and 3 repeat units for mono-, di-, tri-, tetra-, penta-, and hexanucleotide motifs, respectively. Tandem repeats were also verified using the Tandem Repeats Finder (TRF) v4.09 [29] with default parameters.

### 2.3. Phylogenetic and Genetic Distance Analysis

Phylogenetic analysis was conducted using a combined strategy of Bayesian Inference (BI) and Maximum Likelihood (ML) based on the 13 mitochondrial PCGs. In addition to the newly sequenced Oriental Stork (PX682155) mitogenome, we integrated mitogenomic data from the NCBI database for other members of the genus Ciconia, including the Black Stork (*C*. *nigra*, NC_023946.1), the White Stork (*C*. *ciconia*, NC_002197.1), the Oriental Stork (*C. boyciana*, NC_002196), and the Maguari Stork (*C*. *maguari*, MN356211.1). The Crested Ibis (*Nipponia nippon*, NC_008132.1) was selected as the outgroup (Table 1).

The bioinformatics workflow was executed systematically on the PhyloSuite v2.0 [28] platform. Target sequences were retrieved from GenBank, screened, and re-annotated. Multiple sequence alignment was performed using the MAFFT algorithm. For PCGs, MACSE was specifically applied for codon-based alignment to maintain the accuracy of the open reading frames (ORFs). Subsequently, Gblocks was used to remove highly variable or low-quality regions to construct a highly reliable concatenated dataset. During the model selection phase, optimal substitution models were determined based on the Bayesian Information Criterion (BIC) using ModelFinder [30]. For the BI analysis, the HKY+F+I and TN+F+G4 models were selected for *ATP8* and *ND6*, respectively, while the TVM+F+G4 model was applied to the remaining 11 PCGs. For the ML analysis, the HKY+F+I model was selected for *ATP8* and *ND6*, and the GTR+F+G4 model for the other 11 PCGs. Phylogenetic reconstruction was performed using MrBayes 3.2.6 [31] and IQ-TREE 2.0.6 [32]. Branch support was evaluated using Posterior Probabilities (PPs) for BI and Bootstrap Support (BS) for ML, with PP > 0.95 and BS > 70% considered as significant statistical support.

## 3. Results

### 3.1. Characteristics and Composition of the C. boyciana Mitogenome

**Figure 2:**
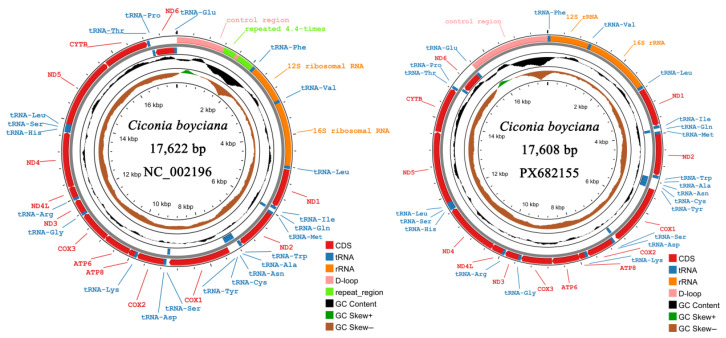
Physical map of the mitochondrial genome of the Oriental Stork (*Ciconia boyciana*). The figure displays the mitogenomic structure of the individual PX682155, with a total length of 17,608 bp. The genome comprises 13 protein-coding genes (PCGs), 22 transfer RNA genes (tRNAs), two ribosomal RNA genes (12S and 16S rRNA), and one non-coding control region (D-loop). Genes located on the outer ring are transcribed on the heavy strand (H-strand), while those on the inner ring are transcribed on the light strand (L-strand).

The mitochondrial genome of the Oriental Stork exhibits a typical circular double-stranded molecular structure (Figure 2). The mitogenomic characteristics of the Oriental Stork (PX682155) were characterized and compared with the reference sequence (NC_002196). Both genomes exhibited a typical vertebrate mitogenome structure, comprising 13 PCGs, two ribosomal RNA genes, 22 transfer RNA genes, and a non-coding control region (D-loop). Detailed genomic features and comparisons are summarized in Table 2. The gene arrangement is highly stable equilibrium between the two sequences, with the primary length variation originating from differences in the number of tandem repeats within the D-loop. Regarding nucleotide composition, both sequences show a significant A+T bias (56.3%), a positive AT-skew (A > T), and a negative GC-skew (C > G). This high consistency in base bias reflects the regular and stable evolutionary selection pressure exerted on the heavy (H) and light (L) strands during mitochondrial DNA replication.

The comparative analysis of the mitogenomic architectures reveals a highly compact structure characteristic of avian mitochondrial genomes. The intergenic regions, comprising both spacers and overlaps, exhibit a high degree of conservation alongside localized variations that reflect subtle spatiotemporal genetic differentiation (Table 2). In the reference sequence (NC_002196), a total of 18 intergenic spacers were identified, ranging from 1 to 17 bp in length, with the largest spacer (17 bp) situated between the *tRNA-Leu2* and *ND1*. In contrast, the PX682155 individual possesses 17 spacers, with significant variations observed in specific regions. Notably, the spacer between *tRNA-Arg* and *ND4L* expanded from 1 bp in the reference to 7 bp in the mainland individual, while the *tRNA-Glu* spacer increased from 2 bp to 3 bp. These expansions contribute to the overall length fluctuations between the two mitogenomes. Gene overlaps, indicative of extreme genomic efficiency, are also prominent. Both individuals share several major overlaps, including the characteristic 10 bp overlap between *ATP8* and *ATP6*, a 7 bp overlap between *ND4L* and *ND4*, and a 9 bp overlap at the *tRNA-Ser2* locus. However, the NC_002196 individual exhibits more frequent small-scale overlaps, such as a 2 bp overlap at *tRNA-Ile* and 1 bp overlap at both *tRNA-Trp* and *COX3*, which are absent in PX682155. Conversely, PX682155 displays unique 1 bp overlap at the *tRNA-Met* and *tRNA-Leu1* positions. Overall, while the core functional regions remain highly synchronized, these differences in intergenic nucleotides—particularly the variations in the *ND4L* spacer and the presence of localized overlaps—serve as molecular evidence of the evolutionary divergence between the East Asian mainland and island lineages.

### 3.2. Sequence Variation and Base Bias in Protein-Coding Genes (PCGs)

**Figure 3:**
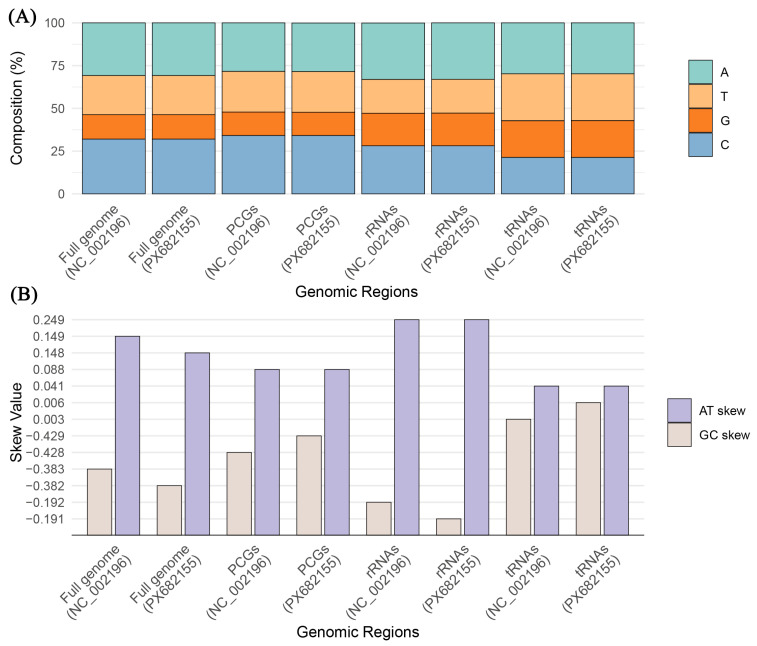
Comparison of nucleotide composition and skewness in the mitochondrial genomes of *Ciconia boyciana* individuals (PX682155 and NC_002196). (**A**) Nucleotide composition percentage across different genomic regions: The stacked bar chart illustrates the relative proportions of Adenine (A), Thymine (T), Guanine (G), and Cytosine (C) in the full genome, PCGs, rRNAs, and tRNAs. Both the 2025 sample (PX682155) and the 2000 reference (NC_002196) display nearly identical composition patterns, characterized by a significant A+T bias. (**B**) AT-skew and GC-skew values across genomic regions: This panel compares the strand-specific nucleotide biases. Positive AT-skew and negative GC-skew values are consistent across all regions for both individuals, reflecting stable evolutionary selection and mutational pressures on the mitochondrial DNA strands over the 25-year span.

Alignment of the PCG regions (Figure 3A,B) shows that while PX682155 and NC_002196 exhibit high evolutionary stability, subtle differences exist. The total PCG length of PX682155 is 11,382 bp, which is 9 bp shorter than the reference (11,391 bp). This variation is entirely due to length changes in genes encoded on the heavy strand (−9 bp), while the light strand remains identical at 522 bp. Ten out of the 13 PCGs are identical in length; differences are localized to *ATP6* (683 bp, −1 bp), *ND1* (976 bp, −2 bp), and *ND2* (1040 bp, −1 bp) (Table 1). These discrepancies likely arise from heterogeneity in start/stop codon recognition or minor codon deletions between individuals. The base composition of PCGs is nearly identical, with an A+T content of 52.2% in both, underscoring high conservation. Minute differences appear in G content and GC bias: PX682155 has a G content of 13.6% (0.1% lower than NC_002196) and a GC-skew of −0.429 (compared to −0.428), indicating a slightly stronger preference for Cytosine (C > G) in the newly sequenced individual.

### 3.3. Codon Usage Bias (RSCU) and Correlation Analysis

**Figure 4:**
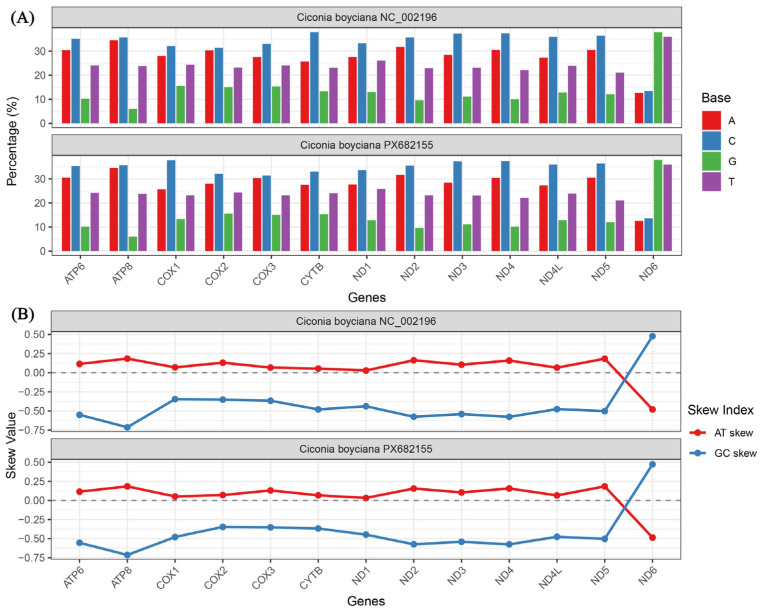
Comparative analysis of mitochondrial protein-coding genes (PCGs) between *Ciconia boyciana* PX682155 and the reference sequence NC_002196. (**A**) Distribution of length variation across the PCG regions of the two sequences. The total length of PCGs in PX682155 is 11,382 bp, which is 9 bp shorter than the reference sequence; the discrepancies are primarily concentrated in the *ATP6*, *ND1*, and *ND2* genes. (**B**) Comparison of base composition bias (GC-skew) in the PCG regions. Both sequences exhibit a significant preference for cytosine (negative GC-skew), reflecting evolutionary stability during mitochondrial DNA replication.

Analysis of the 13 PCGs demonstrates high homogeneity in most genes (Figure 4A,B). All 13 PCGs, including *COX1* (1551 bp), exhibit identical lengths between the two individuals, with the exception of minor 1–2 bp variations in *ATP6*, *ND1*, and *ND2*. Nucleotide content and skewness (AT-skew, GC-skew) are extremely similar; for instance, *ATP8* values are perfectly matched between sequences. Notably, *ND6*, the only gene encoded on the L-strand, exhibits a consistent reverse bias (AT-skew < 0, GC-skew > 0) in both sequences, reflecting strand-specific selection. The PCGs exhibit highly stable start and stop codon strategies. Except for *COX1* and *COX2*, which use GTG, all genes initiate with ATG (Table 1). Termination codons include standard forms as well as incomplete (T, TA) and special (AGG) types: *ATP6* and *ND2* use TA; *ND1*, *ND4*, and *COX3* use T; and *ND1*, *ND5*, and *COX1* recognize AGG as a stop signal.

**Figure 5:**
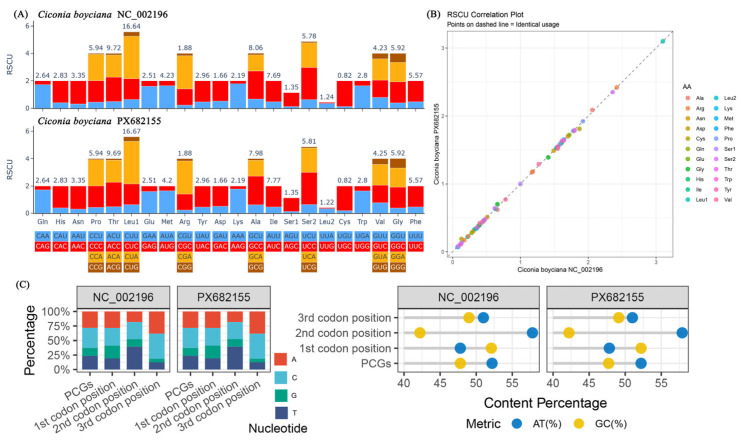
Analysis of codon usage bias (RSCU) and nucleotide composition in the mitochondrial genome of the Oriental Stork (*Ciconia boyciana*). (**A**) Distribution of RSCU frequencies for the 20 amino acids. The stacked bar charts illustrate the usage preference for different codons, with Leucine (Leu1) exhibiting the highest frequency (~16.6%) and a notable preference for codons ending in A or C. (**B**) Linear correlation analysis of RSCU values between PX682155 and the reference sequence NC_002196. Each scatter point represents a specific synonymous codon. All points are tightly clustered along the diagonal dashed line (Identical usage line), demonstrating the high conservation of translational pressure within the species. (**C**) Nucleotide composition (left) and AT/GC content (right) across codon positions: This panel summarizes the nucleotide distributions and AT/GC percentages for the entire PCGs and at each codon position (1st, 2nd, and 3rd) for PX682155 and NC_002196. The results show highly synchronized base distributions between the two sequences, characterized by a significant enrichment of Cytosine (C) and a deficiency of Guanine (G) at the third position. Furthermore, the AT content at the second position is notably higher than the GC content, likely reflecting functional constraints for encoding hydrophobic amino acids. The nearly identical distribution patterns between the 2000 (NC_002196) and 2025 (PX682155) samples underscore the high evolutionary stability of the Ciconia boyciana mitogenome over the past 25 years.

RSCU analysis (Figure 5A) shows that Leucine (Leu1), Threonine (Thr), and Alanine (Ala) are the most frequent, with a clear preference for codons ending in A or C. Correlation analysis (Figure 5B) confirms that the RSCU values of PX682155 and the reference are nearly identical, with all points clustering tightly along the diagonal, proving stable selection and mutational pressures within the species. Independent analysis of the three codon positions (Figure 5C) shows synchronized patterns. The GC content at the first position (1st) is consistently maintained at 52.2%, reflecting strong functional constraints. The second position (2nd) is characterized by high T content (~39.6%), correlating with the dominance of hydrophobic amino acids, and shows strong purifying selection. The third position (3rd) is the most variable, with an extreme bias (C content 43.2% vs. G content 5.9%). The synchronized GC-skew (−0.760) at the third position reflects similar intense mutational or selection pressures across the 25-year span.

### 3.4. Transfer RNA (tRNAs) and Ribosomal RNA (rRNAs)

Both sequences contain 22 tRNAs and two rRNAs in an identical arrangement, with no gene rearrangements detected (Table 2). The rRNAs exhibit a consistent A+T preference (52.8%) and a stable AT-skew (0.249) (Figure 3A,B). The 22 tRNAs are highly synchronized in length and secondary structure, with an A+T content of ~57.3% and a GC-skew (~0.003) near zero. This stability indicates that non-coding RNA regions are subject to stringent functional constraints and have remained structurally consistent between the 2000 and 2025 samples.

### 3.5. Non-Uniformity and Polymorphism in the Control Region (D-Loop)

While the D-loop regions of PX682155 and NC_002196 are highly homologous, significant polymorphisms exist. The 5′ end is relatively well-preserved but contains several Single Nucleotide Polymorphisms (SNPs), including transitions (e.g., A to G or T to C). The central domain contains multiple tandem repeat units (e.g., “GTTTG…AAACAC”). The most striking differences occur in the 3′ microsatellite region composed of “ACAA” or “CAA” simple sequence repeats (SSRs). Length polymorphisms in these SSRs account for the overall physical length discrepancy between the two D-loop sequences. Despite this hypervariability caused by replication slippage, the functional framework (ending in “ACAGCCTCAACC”) remains stable within the species.

The nucleotide composition of the D-loop was also compared: PX682155 (2053 bp; A: 31.4%, T: 24.8%, C: 29.5%, G: 14.3%) and NC_002196 (2039 bp; A: 31.2%, T: 25.1%, C: 29.3%, G: 14.4%). Despite its hypervariable nature in terms of length, the overall base proportions in the D-loop remain relatively stable between the two individuals.

### 3.6. Phylogenetic Analysis

**Figure 6:**
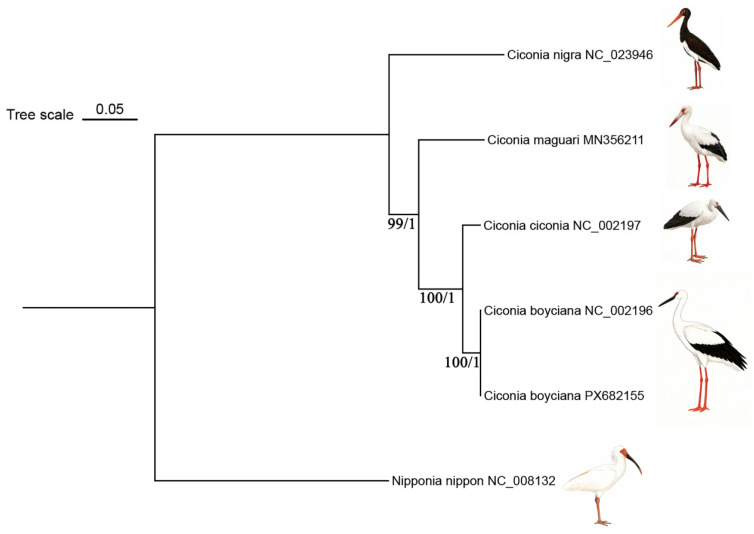
Phylogenetic tree of Ciconiidae species reconstructed based on complete mitochondrial genome sequences. The molecular phylogenetic tree was constructed using Maximum Likelihood (ML) and Bayesian Inference (BI) methods. Numbers at the nodes represent Bootstrap support (BS) and Posterior probability (PP), respectively.

A phylogenetic tree was reconstructed using the concatenated nucleotide sequences of the 13 PCGs via ML and BI) Both methods yielded identical topologies (Figure 6), confirming the reliability of the classification. The genus *Ciconia* forms a distinct monophyletic clade. Within this clade, the Oriental Stork is closest to the White Stork, forming a sister-group relationship, while phylogenetic distances to the Maguari Stork and the Black Stork increase sequentially.

## 4. Discussion

In this study, we successfully assembled the complete mitochondrial genome of an Oriental Stork from Hongze Lake, Jiangsu. Its total length of 17,608 bp aligns closely with the typical mitochondrial structure observed in vertebrates [33,34]. Our findings reveal that the mainland Chinese individual (PX682155) and the Japanese reference individual (NC_002196), despite being separated by a 25-year spatiotemporal gap, exhibit extreme homogeneity in gene arrangement, base bias, and PCG composition [23]. This high degree of genomic conservation is consistent with strong functional constraints typically imposed on mitochondrial coding regions. Such patterns align with the expected outcomes of purifying selection, which acts to preserve the physiological integrity of the oxidative phosphorylation system despite historical population bottlenecks [10,35]. Specifically, the highly synchronized nucleotide substitution patterns across the three codon positions of the PCGs further corroborate a relatively stable evolutionary rate at the molecular level for this species. The extreme Cytosine (C) enrichment and significant negative GC-skew (GC-skew = −0.760) at the third codon position are likely shaped by combined mutational and selective pressures. Our comparative analysis with other Ciconiidae species (such as *C. nigra*) reveals a consistent trend of C-bias and negative GC-skew at the third position [34], suggesting that this bias is a conserved genomic feature across the family rather than a species-specific trait of *C. boyciana*. This reflects the inherent replicative asymmetry and biased mutation pressure common in avian mitogenomes, particularly at the relatively degenerate third codon position [36].

Despite the overall structural conservation of the mitogenome, comparison of the D-loop control regions reveals significant length polymorphism, primarily stemming from replication slippage of tandem repeats [37]. The differences between PX682155 and the Japanese individual are concentrated in the “ACAA/CAA” microsatellite repeat region, suggesting a long-term lack of gene flow. This likely reflects a historical geographic isolation between the East Asian mainland populations and the Japanese reintroduced populations (which largely originated from the Russian Far East). Minor variations in the total length of the mitogenome are common among different individuals of the Oriental Stork. Previous studies have shown that these length changes are primarily localized in the non-coding D-loop, particularly within two types of tandem repeats at its 3′ end: Repeat A (a 71 bp direct repeat) and Repeat B (long CAAA repeats). As reported in previous research [23], the Repeat B region exhibits significant heteroplasmy, with lengths varying from 323 bp to 423 bp across different individuals or even different clones within the same individual. In this study, the D-loop length of PX682155 was determined to be 2039 bp, which is slightly shorter than the reference sequences (2053 bp) but remains within the range of individual variation documented in the literature. Given that the D-loop is the most hypervariable region in mtDNA, its length and haplotype diversity provide crucial genetic information for monitoring and avoiding inbreeding in captive populations. Furthermore, consistent with previous studies [38,39], our phylogenetic analyses confirm the monophyly of *C. boyciana* and its sister-group relationship with *C. ciconia*. The addition of the PX682155 mitogenome enriches the genetic database for this endangered species, providing essential data for future population genomics research and the refinement of conservation strategies.

However, it must be acknowledged that this study is based on a comparison between only two individual mitogenomes. While these results provide a valuable temporal snapshot, they represent an individual-level comparison rather than a comprehensive assessment of population-level genetic diversity or spatiotemporal dynamics. Therefore, the observed homogeneity and D-loop variations should be interpreted as preliminary insights. To fully distinguish individual variation from broader population patterns, further research involving a larger sample size from diverse geographic regions and multiple time points is essential. Future multi-sample studies from diverse EAAF sites will validate population-level patterns.

In summary, this study successfully assembled and characterized the complete mitochondrial genome of a contemporary Oriental Stork individual from Hongze Lake (2025), providing a vital genomic benchmark for the sampled individual from the East Asian mainland. Our comparative analysis reveals a striking degree of structural and functional conservation in protein-coding genes over a 25-year spatiotemporal span, suggesting that the species has maintained high metabolic efficiency and evolutionary stability despite historical population bottlenecks. While the core genome remains highly constrained under purifying selection, significant length polymorphisms identified within the D-loop control region potentially serve as molecular markers for investigating geographic isolation [40], though more samples are needed to confirm these patterns. While our preliminary findings are consistent with the idea that recent “southward expansion” and behavioral shift toward sedentary breeding in the Yangtze River basin might be driven by phenotypic plasticity, this hypothesis requires verification through large-scale genomic screening within a stable genetic framework rather than large-scale genomic reorganization. By bridging the gap in mitogenomic data between island and continental individuals, this research offers a refined scientific basis for monitoring genetic diversity and formulating targeted ex situ conservation strategies [41]. Ultimately, these molecular insights are indispensable for ensuring the long-term resilience of the Oriental Stork as it adapts to the evolving wetland landscapes of the East Asian–Australasian Flyway.

## 5. Conclusions

In conclusion, this study successfully assembled and characterized the complete mitogenome of a 2025 Oriental Stork (*C. boyciana*) from Hongze Lake, providing a contemporary genomic benchmark for the mainland population. Our comparative analysis indicates high structural and functional conservation in protein-coding genes over a 25-year spatiotemporal span, while identifying significant length polymorphisms within the D-loop region. These findings establish the D-loop as a sensitive molecular marker for monitoring lineage differentiation and genetic diversity. This research bridges the data gap between island and continental populations, offering a scientific basis for formulating targeted ex situ conservation and management strategies for this endangered species within the East Asian–Australasian Flyway.

## Figures and Tables

**Table 1:** The species used for the phylogenetic analysis in this study.

Family	Organism	Length (bp)	AT Skew	GC Skew	AT%	GC%	GenBank ID
Ciconiidae	*Ciconia boyciana*	17,608	0.148	−0.382	53.7	46.3	PX682155 (provided in this study)
Ciconiidae	*Ciconia boyciana*	17,622	0.149	−0.383	53.7	46.3	NC_002196 (*Ciconia boyciana* mitochondrion, complete genome—Nucleotide—NCBI)
Ciconiidae	*Ciconia nigra*	17,795	0.133	−0.382	54.3	45.7	NC_023946 (*Ciconia nigra* mitochondrion, complete genome—Nucleotide—NCBI)
Ciconiidae	*Ciconia ciconia*	17,347	0.138	−0.380	53.6	46.4	NC_002197 (*Ciconia ciconia* mitochondrion, complete genome—Nucleotide—NCBI)
Ciconiidae	*Ciconia maguari*	17,431	0.122	−0.376	54.0	46.0	MN356211 (*Ciconia maguari* mitochondrion, partial genome—Nucleotide—NCBI)
Threskiornithidae	*Nipponia nippon*	16,732	0.129	−0.381	53.9	46.1	NC_008132 (*Nipponia nippon* mitochondrion, complete genome—Nucleotide—NCBI)

**Table 2:** Mitogenomic characteristics of *Ciconia boyciana* PX682155 and NC_002196. Values separated by a slash (/) represent data for NC_002196 (left) and PX682155 (right), respectively. In the “Intergenic Nucleotides” column, positive values indicate the number of nucleotides in the intergenic spacer between two adjacent genes; negative values indicate the number of overlapping nucleotides between genes. For example, “−10” denotes a 10 bp gene overlap.

Gene	Position		Size	Intergenic Nucleotides	Codon		Strand
	From	To			Start	Stop	
*tRNA-Phe*	2054/1	2123/70	70/70				H
*12S rRNA*	2124/71	3091/1038	968/968				H
*tRNA-Val*	3092/1039	3162/1109	71/71				H
*16S rRNA*	3163/1110	4774/2721	1612/1612				H
*tRNA-Leu2*	4775/2722	4848/2795	74/74				H
*ND1*	4866/2813	5843/3788	978/976	17/17	ATG/ATG	AGG/T	H
*tRNA-Ile*	5842/3789	5913/3860	72/72	−2/—			H
*tRNA-Gln*	5922/3869	5992/3939	71/71	8/8			L
*tRNA-Met*	5993/3939	6060/4007	68/68	—/−1			H
*ND2*	6061/4008	7101/5047	1041/1040		ATG/ATG	TAA/TA	H
*tRNA-Trp*	7101/5048	7176/5123	76/76	−1/—			H
*tRNA-Ala*	7178/5125	7246/5193	69/69	1/1			L
*tRNA-Asn*	7249/5196	7321/5268	73/73	2/2			L
*tRNA-Cys*	7324/5271	7390/5337	67/67	2/2			L
*tRNA-Tyr*	7391/5338	7460/5407	70/70				L
*COX1*	7462/5409	9012/6959	1551/1551	1/1	GTG/GTG	AGG/AGG	H
*tRNA-Ser2*	9004/6951	9077/7024	74/74	−9/−9			L
*tRNA-Asp*	9080/7027	9148/7095	69/69	2/2			H
*COX2*	9150/7097	9833/7780	684/684	1/1	GTG/GTG	TAA/TAA	H
*tRNA-Lys*	9835/7782	9906/7853	72/72	1/1			H
*ATP8*	9908/7855	10,075/8022	168/168	1/1	ATG/ATG	TAA/TAA	H
*ATP6*	10,066/8013	10,749/8695	684/683	−10/−10	ATG/ATG	TAA/TA	H
*COX3*	10,749/8696	11,532/9479	784/784	−1/—	ATG/ATG	T/T	H
*tRNA-Gly*	11,533/9480	11,601/9548	69/69				H
*ND3*	11,602/9549	11,952/9899	351/351		ATG/ATG	TAA/TAA	H
*tRNA-Arg*	11,957/9904	12,025/9972	69/69	4/4			H
*ND4L*	12,027/9974	12,323/10,270	297/297	1/7	ATG/ATG	TAA/TAA	H
*ND4*	12,317/10,264	13,694/11,641	1378/1378	−7/−7	ATG/ATG	T/T	H
*tRNA-His*	13,695/11,642	13,764/11,711	70/70				H
*tRNA-Ser1*	13,766/11,712	13,829/11,777	64/64	1/—			H
*tRNA-Leu1*	13,830/11,777	13,900/11,847	71/71	—/−1			H
*ND5*	13,901/11,848	15,712/13,659	1812/1812		ATG/ATG	AGG/AGG	H
*CYTB*	15,723/13,670	16,865/14,812	1143/1143	10/10	ATG/ATG	TAA/TAA	H
*tRNA-Thr*	16,867/14,814	16,934/14,881	68/68	1/1			H
*tRNA-Pro*	16,946/14,893	17,015/14,962	70/70	11/11			L
*ND6*	17,026/14,973	17,547/15,494	522/522	10/10	ATG/ATG	TAG/TAG	L
*tRNA-Glu*	17,550/15,498	17,622/15,569	73/72	2/3			L
D-loop	1/15,570	2053/17,608	2053/2039				

## Data Availability

The data presented in this study were deposited in the NCBI repository under accession number PX682155.

## Abbreviations

The following abbreviations are used in this manuscript:

ML	Maximum Likelihood
BI	Bayesian Inference
rRNA	ribosomal RNA genes
tRNA	transfer RNA genes
PCGs	Protein-coding genes
RSCU	Relative Synonymous Codon Usage
SSR	The microsatellite repeat sequences
EAAF	East Asian–Australasian Flyway
SNPs	Single Nucleotide Polymorphisms
